# Prevalence and clinical significance of lumbosacral transitional vertebra (LSTV) in a young back pain population with suspected axial spondyloarthritis: results of the SPondyloArthritis Caught Early (SPACE) cohort

**DOI:** 10.1007/s00256-017-2581-1

**Published:** 2017-02-24

**Authors:** F. de Bruin, S. ter Horst, J. L. Bloem, R. van den Berg, M. de Hooge, F. van Gaalen, H. Dagfinrud, M. van Oosterhout, R. Landewé, D. van der Heijde, M. Reijnierse

**Affiliations:** 1 Department of Radiology, Leiden University Medical Center, 2333 ZA Leiden, The Netherlands; 2 Department of Rheumatology, Leiden University Medical Center, Leiden, The Netherlands; 3 Department of Rheumatology, Diakonhjemmet Hospital, Oslo, Norway; 4 Department of Rheumatology, Amsterdam Medical Center, Amsterdam, The Netherlands; 5 Department of Rheumatology, Groene Hartziekenhuis, Gouda, The Netherlands

**Keywords:** Axial spondyloarthritis, Lumbosacral transitional vertebra, Disc degeneration

## Abstract

**Objective:**

To determine in a cohort of young patients with suspected axial spondyloarthritis (axSpA), the prevalence of lumbosacral transitional vertebra (LSTV), its association with local bone marrow edema (BME) and lumbar spine degeneration, and the potential relationship with MRI findings and clinical signs of axSpA.

**Materials and methods:**

Baseline imaging studies and clinical information of patients from the SPondyloArthritis Caught Early-cohort (back pain ≥3 months, ≤2 years, onset <45 years) were used. Two independent readers assessed all patients for LSTV on radiography, and BME-like and degenerative changes on MRI. Patients with and without LSTV were compared with regard to the prevalence of MRI findings and the results of clinical assessment using Chi-squared test or *t* test.

**Results:**

Of 273 patients (35.1% male, mean age 30.0), 68 (25%) patients showed an LSTV, without statistical significant difference between patients with and without axSpA (*p* = 0.327). Local sacral BME was present in 9 out of 68 (13%) patients with LSTV and absent in patients without LSTV (*p* < 0.001). Visual analogue scale (VAS) pain score and spinal mobility assessments were comparable.

**Conclusions:**

LSTV is of low clinical relevance in the early diagnosis of axSpA. There is no difference between patients with and without LSTV regarding the prevalence of axSpA, pain and spinal mobility, and a BME-like pattern at the pseudoarticulation does not reach the SI joints.

## Introduction

Spondyloarthritis (SpA) is a chronic inflammatory rheumatic disease, either predominantly in the spine (axial spondyloarthritis; axSpA) or in the peripheral joints (peripheral SpA). AxSpA is characterized by chronic low back pain, often associated with morning stiffness. Bone marrow edema-like changes (BME) in the sacroiliac joints are of interest in axSpA, as the Assessment of Spondyloarthritis International Society (ASAS) defined sacroiliitis on magnetic resonance imaging (MRI) as part of the classification criteria, in addition to sacroiliitis on radiography and a complex of clinical and laboratory parameters, the so-called SpA features [1, 2].

**Fig. 1:**
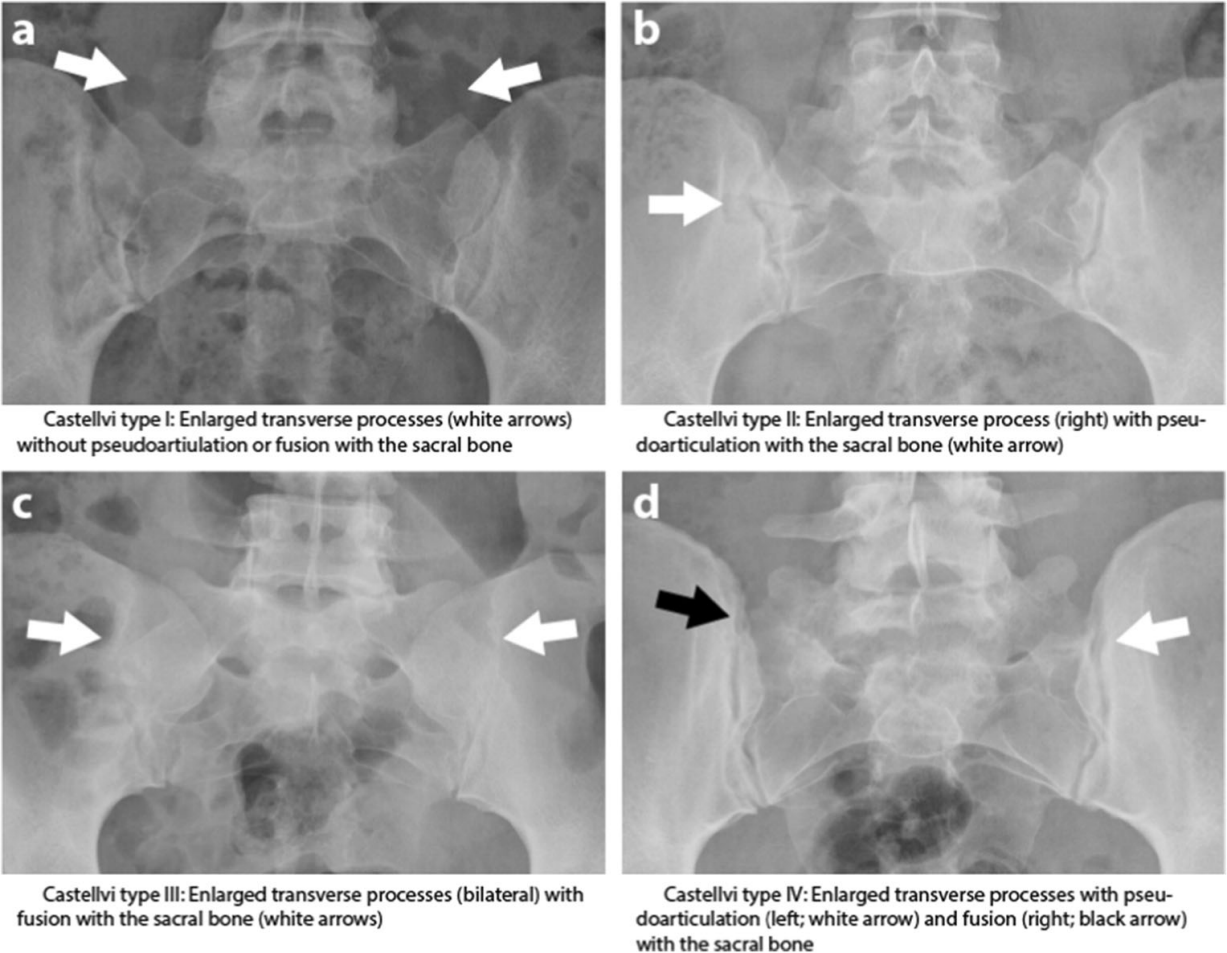
Radiographs of the Castellvi classification of lumbosacral transitional vertebra. **a** Type I: enlarged transverse processes (*white arrows*) without pseudoarticulation or fusion with the sacral bone. **b** Type II: enlarged transverse process (*right*) with pseudoarticulation with the sacral bone (*white arrow*). c Type III: enlarged transverse processes (bilateral) with fusion with the sacral bone (*white arrows*). **d** Type IV: enlarged transverse processes with pseudoarticulation (*left*; *white arrow*) and fusion (*right*; *black arrow*) with the sacral bone

Many patients with suspected axSpA are screened with standard anteroposterior (AP) radiographs of the pelvis for sacroiliitis and to rule out other possible causes of back pain. The lumbosacral transitional vertebra (LSTV) is a congenital anomaly of the lumbosacral transition, in which the transverse process of the last lumbar vertebra is enlarged, either unilaterally or bilaterally [3]. Castellvi et al. categorized the LSTV according to the interaction of the transverse process to the sacral/iliac bone: enlarged transverse process without interaction (grade I), pseudo-articulation (grade II), fusion (grade III), and articulation and fusion (grade IV; Fig. 1) [4].

The prevalence of LSTV in the general population has been reported to be between 16 and 36% [5, 6], and some studies have suggested an association between LSTV and lower back pain (LBP) [5, 7, 8].

We hypothesized that, because of alterations in the lumbosacral anatomy associated with an LSTV, the biomechanics of the pelvic region might be altered, mimicking MRI findings and clinical symptoms of axSpA. Therefore, we determined in a cohort of young patients with suspected axSpA, the prevalence of LSTV, its association with BME and lumbar spine degeneration, and the potential relationship with MRI findings and clinical signs of axSpA.

## Patients and methods

Patients from the SpondyloArthritis Caught Early (SPACE) cohort, included between January 2009 and December 2012, were assessed [9]. The SPACE cohort is an ongoing multicenter prospective observational study. Inclusion criteria are chronic (almost daily) back pain for longer than 3 months, but no longer than 2 years with the onset before the 45th year. Exclusion criteria were age <16 years, other known painful conditions not related to axSpA, and any other reason that could interfere with disease evaluation, signing informed consent and/or compliance with the protocol. The local medical ethics committees of the participating centers approved the research protocol for the SPACE cohort and written informed consent of all patients was obtained. Patients were categorized into three groups based on the ASAS axSpA criteria (ASAS status): no-axSpA (not fulfilling ASAS axSpA criteria), possible axSpA (not fulfilling ASAS axSpA criteria but ≥1 high specific axSpA feature (human leukocyte antigen [HLA]-B27, positive family history for SpA, sacroiliitis on radiographs and/or MRI, acute anterior uveitis) or ≥2 less specific axSpA features (inflammatory back pain, enthesitis, peripheral arthritis, psoriasis, inflammatory bowel disease, good response to NSAIDs, elevated levels of CRP) or definite axSpA (fulfilling ASAS axSpA criteria).

At baseline, the 10-cm modified Schober’s test and lateral spinal flexion test were performed [10, 11]. The mean score of the left and right lateral spinal flexion test was available. Patients were asked in which part of the spine they experienced pain—thoracic, lumbar, buttock or a combination of these locations—and reported the intensity of the pain on a visual analogue scale (VAS; from 0, no pain, to 10, unbearable pain). Patients were categorized as having LBP if they experienced lumbar and/or buttock pain.

### Imaging technique

The SPACE protocol includes sagittal T1-weighted turbo spin echo (T1TSE; repetition time [TR)] 550 ms/echo time [TE] 10 ms) and short tau inversion recovery (STIR; TR 2,500 ms/TE 60 ms) sequences of the entire (cervical, thoracic and lumbar) spine and coronal oblique images of the sacroiliac (SI) joints. MRI was performed on a 1.5 T-MR system and the slice thickness was 4 mm. In addition, lateral radiographs of the lumbar spine and AP radiographs of the pelvis were obtained.

### Radiological data

Two readers independently scored the images, blinded for patient characteristics, clinical outcome and the other imaging studies.

Lumbosacral transitional vertebra was assessed according to the Castellvi classification (Fig. 1) [4] on AP pelvic radiographs, patients without an LSTV are referred to as no-LSTV patients and patients with an LSTV (regardless of which type) are referred to as LSTV patients. Disc degeneration (Pfirrmann classification) [12], disc herniation [13] and end plate changes (Modic classification) [14] were scored on lumbar STIR and T1-weighted MRI. Disc degeneration was defined as a Pfirrmann class of 3 or higher. BME at the superior border of the sacrum and the transverse processes was assessed on STIR MR SI images (referred to as “local BME”). In the case of disagreement between the readers, adjudication was performed on a per lesion basis, except for disc degeneration where adjudication was performed only when the difference between readers was two Pfirrmann grades or more. In the case of disagreement of 1 grade, the lowest grade was used. Two other readers independently scored the SI joints as a whole (according to the ASAS definition) and per quadrant (with the Spondyloarthritis Research Consortium of Canada [SPARCC] method) for the presence (yes/no) of inflammatory lesions associated with axSpA. ASAS definition: positive when ≥2 inflammatory lesions highly suggestive of axSPA on one slice or one lesion seen on ≥2 consecutive slides [15]. SPARCC method: the presence of inflammation is scored as present/absent in each quadrant of the SI joint in 6 consecutive slices [16]. Only a quadrant with two or more consecutive slices with BME was considered positive for inflammation (i.e. associated with axSpA) in the current analysis. These readers were also blinded to clinical information and the scores of the other reader. An adjudicator was introduced if the readers disagreed on the ASAS definition.

### Statistics

All the scores presented are based on adjudicated scores. Baseline characteristics are presented for patients with and without LSTV. Categorical data are reported using frequencies and percentages. Continuous data are reported with mean and range or standard deviation (SD). Student’s *t* test and Chi-squared test were used to assess differences between patients with and without LSTV for continuous data and categorical data respectively. Kappa values reported were calculated according to Landis and Koch [17].

## Results

Two hundred and seventy-three patients of the SPACE cohort with complete imaging and clinical information were used, mean age was 30.0 years (range 16–45) and 96 (35.1%) were men. Of the 273 patients, 27 (9.9%) were categorized as no-axSpA, 134 (49.1%) as possible axSpA and 112 (41.0%) as axSpA. An LSTV was found in 68 out of 273 patients (24.9%). Of these, 35 out of 68 (51.5%) were Castellvi type I, 11 out of 68 (16.2%) type II (4 of which were unilateral), 17 out of 68 (25.0%) type III (1 unilateral) and 5 out of 68 (7.4%) type IV. Table 1 presents the baseline characteristics of LSTV and non-LSTV groups.

**Table 1:** Baseline characteristics of patients with and without LSTV

	No-LSTV (*n* = 205)	LSTV (*n* = 68)	*p* value
Age at inclusion, mean (SD)	29.7 (8.4)	31.0 (7.5)	0.22
Male, *n* (%)	64 (31.2)	31 (45.6)	0.086
ASAS axSpA criteria status			0.327*
No axSpA (*n* = 27), *n* (%)	23 (85.2)	4 (14.8)	
Possible axSpA (*n* = 134), *n* (%)	102 (76.1)	32 (23.9)	
AxSpA (*n* = 112), *n* (%)	80 (71.4)	32 (28.6)	
HLA-B27 positive, *n* (%)	69 (34)	26 (38)	0.712
Sacroiliitis on MRI, *n* (%)	40 (20)	18 (26)	0.362

*LSTV* lumbosacral transitional vertebra, *ASAS *Assessment of Spondyloarthritis International Society, *axSpA* axial spondyloarthritis, *HLA-B27* human leukocyte antigen B27, *MRI* magnetic resonance imaging

*Chi-squared test comparing ASAS axSpA criteria status and presence of LSTV

### Radiological findings

Weighted kappa for inter-reader agreement was substantial (κ = 0.61). Local BME was present in none of the no-LSTV patients and present in 9 out of 68 (13.0%) LSTV patients (*p* < 0.001). Of these 9 patients, 4.5% (5 out of 112) were catagorized as axSpA and 2.5% as either no-axSpA or possible axSpA (4 out of 158). Relative risk of axSpA based on the presence of BME was 1.38 with 95%CI 0.76–2.52 (*p *= 0.49).

On the lumbar spine MRI, disc degeneration, herniation and Modic changes were more prevalent in L5–S1 than in L4–L5 in no-LSTV patients (details are listed in Table 2). In contrast, for LSTV patients, degeneration was similarly or less prevalent in L5–S1 than in L4–L5. No significant differences between patients with and without LSTV were found for any of the degenerative changes on both L4–L5 and L5–S1. Details for LSTV subtypes are shown in Table 2.

**Table 2:** Degenerative and axial spondyloarthritis-associated radiological findings in the spine and SI joints in no-LSTV patients and LSTV patients

	No LSTV (*n* =205)	All types LSTV (*n* = 68)	Type I LSTV (*n* = 35)	Type II LSTV (*n* = 11)	Type III LSTV (*n* = 17)	Type IV LSTV (*n* = 5)	*p* value*
Spine							
Degeneration of the second last lumbar vertebral unit, *n* (%)							
Disc degeneration	50 (24)	17 (25)	8 (23)	2 (18)	5 (29)	2 (40)	0.993
Modic changes	10 (5)	7 (10)	4 (11)	2 (18)	0	1 (20)	0.118
Herniation	40 (20)	12 (18)	3 (9)	3 (27)	4 (24)	2 (40)	0.700
Degeneration of the last lumbar vertebral unit, *n* (%)							
Disc degeneration	66 (32)	16 (24)	8 (23)	1 (9)	5 (29)	2 (40)	0.229
Modic changes	17 (8)	2 (3)	2 (6)	0	0	0	0.121
Herniation	52 (25)	11 (16)	5 (14)	0	5 (29)	1 (20)	0.176
SI joints							
BME at the site of (pseudo-) articulation, *n* (%)	0	9 (13)	3 (8)	4 (36)	2 (12)	1 (20)	<0.001
Inflammation associated with axSpA in one or both upper quadrants of the SI joints^a^, *n* (%)	22 (10.4)	11 (16.2)	5 (14.3)	3 (27.3)	3 (17.6)	0	0.328
Inflammation associated with axSpA^b^, *n* (%)	15 (7.3)	10 (14.7)	6 (17.1)	2 (18.2)	2 (11.8)	0	0.112

*BME* bone marrow edema-like lesions

*p values for comparing the no-LSTV group with the LSTV group

^a^Scored according to the Spondyloarthritis Research Consortium of Canada method

^b^Scored according to the Assessment of Spondyloarthritis International Society definition

When comparing no-LSTV and LSTV patients for the presence of inflammatory lesions associated with axSpA, we found no difference for inflammatory lesions in the upper quadrants: 22 out of 205 no-LSTV patients (10.4%) had inflammatory lesions in the upper quadrants and 11 out of 68 LSTV patients (16.2%; *p* = 0.328). For inflammation in the whole SI joints, we found that 15 out of 205 no-LSTV patients (7.3%) and 10 out of 68 LSTV patients (14.7%) were positive; there was also no statistically significant difference (*p* = 0.112). For details of the LSTV subgroups, see Table 2.

### Clinical parameters

In 27 patients without axSpA, 2 patients (6.7%) were categorized as an LSTV type I, 1 (3.3%) with type III and 1 (3.3%) with type IV. In 134 patients with possible axSpA, 17 (12.7%) were scored with an LSTV type I, 7 (5.2%) with type II, 6 (4.5%) with type III and 2 (1.5%) with type IV. In 112 patients categorized as axSpA, 16 (14.3%) had an LSTV type I, 4 (3.6%) had type II, 10 (8.9%) had type III and 2 (1.8%) had type IV (*p* = 0.327 for the difference among no-axSpA, possible SpA and axSpA). Patients with local edema at the site of the LSTV were evenly distributed among the no-axSpA, possible axSpA and axSpA groups. Results of pain questionnaires and clinical tests were comparable in no-LSTV and LSTV patients (Table 3). LBP was reported by 171 out of 205 no-LSTV patients (88.6%) and by 61 out of 68 LSTV patients (90%; *p* = 0.547). Mean (SD) VAS pain in no LSTV patients was 4.8 (2.3) cm and for LSTV patients 4.8 (2.5) cm (*p* = 0.956). No-LSTV patients had a worse modified Schober’s test (5.0 [1.8] cm) than LSTV patients (5.4 [2.2] cm; *p* = 0.144). Mean lateral spinal flexion test was 17.6 (5.0) cm in no-LSTV patients and 17.7 (4.8) cm in LSTV patients (*p* = 0.838).

**Table 3:** Outcome of clinical assessments and pain questionnaires at baseline for the no-LSTV group and LSTV groups

	No-LSTV (*n* = 205)	All types LSTV (*n* = 68)	Type I LSTV (*n* = 35)	Type II LSTV (*n* = 11)	Type III LSTV (*n* = 17)	Type IV LSTV (*n* = 5)	*p* value*
Lower back pain^a^, *n* (%)	171 (88.6)	61 (90)	30 (85.7)	11 (100)	15 (93.8)	5 (100)	0.547
VAS score, mean (SD)	4.8 (2.3)	4.8 (2.5)	4.9 (2.4)	4.6 (2.7)	4.3 (2.4)	7.0 (2.4)	0.956
Modified Schober’s test in cm, mean (SD)	5.0 (1.8)	5.4 (2.2)	5.1 (1.8)	5.2 (1.2)	5.5 (2.5)	7.4 (4.25)	0.144
Lateral spinal flexion in cm, mean (SD)	17.6 (5.0)	17.7 (4.8)	17.6 (4.7)	17.1 (4.2)	18.2 (5.0)	17.6 (7.3)	0.838

*VAS* visual analogue scale, *LSTV* lumbosacral transitional vertebra

*p values for comparing the no-LSTV group with the LSTV group

^a^Lower back pain: pain in either the lumbar spine or the buttock or both, as indicated by the patient (yes/no)

## Discussion

We found that LSTV does not have an impact on radiological, MRI and/or clinical signs of axSpA. LSTV had a high prevalence (25%) in patients from the SPACE cohort, which is in accordance with the literature [5, 6]. Nine of the sixty-eight LSTV patients (13%) had accompanying local edema (3% of the whole cohort); however, this was not observed in no-LSTV patients. ASAS classification (no-axSpA, possible axSpA, axSpA) was not associated with the presence or absence of LSTV.

**Fig. 2:**
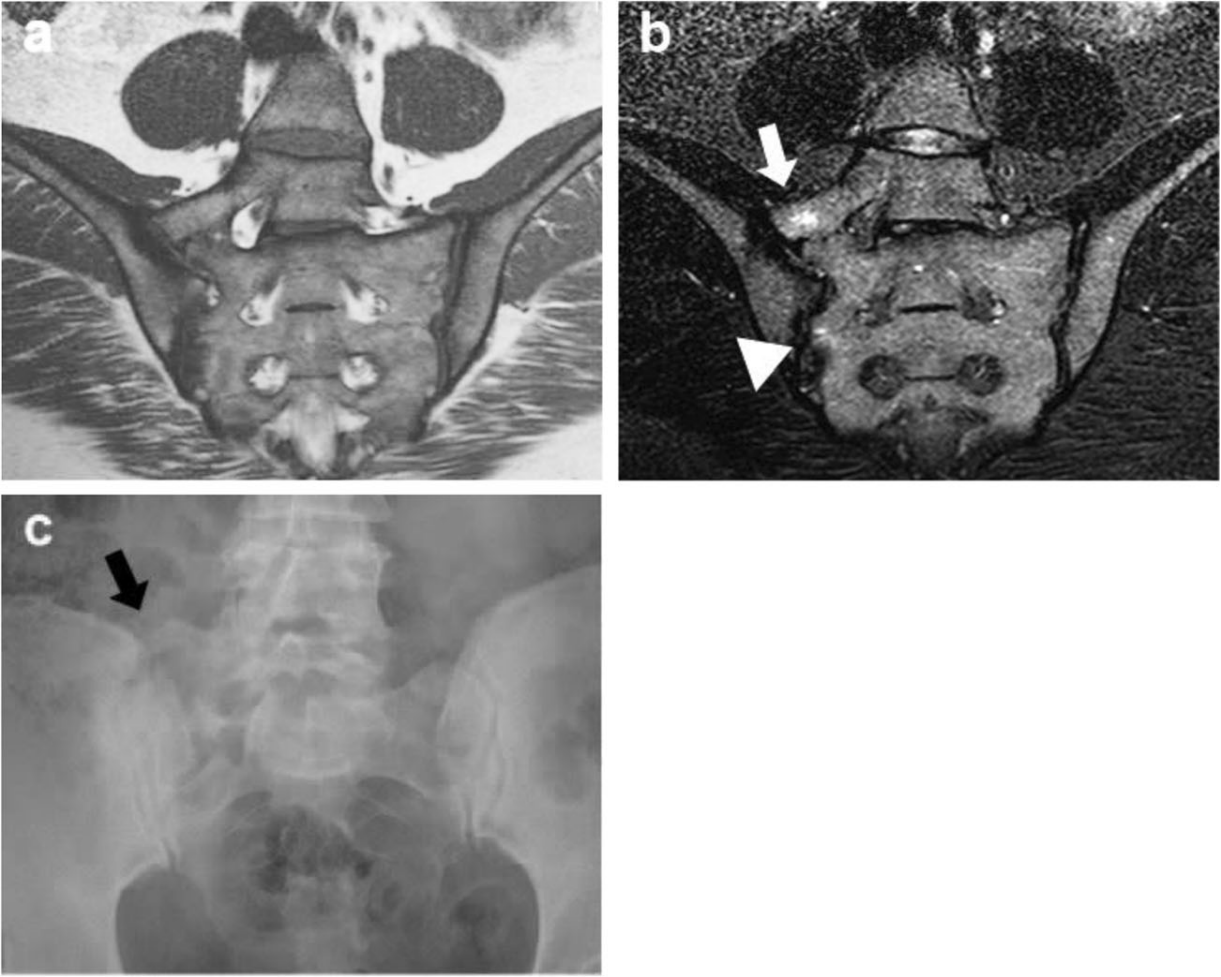
Example of a patient with local edema at the pseudoarticulation site. **a** T1-weighted, **b** STIR and **c** anteroposterior radiograph of the sacroiliac joints in a 38-year-old woman with axial spondyloarthritis. **a** Pseudoarticulation. **b** Bone marrow edema is visible at the site of the pseudoarticulation (*white arrow*), without reaching the sacroiliac joint. In addition, subtle bone marrow is visible in the lower half of the sacroiliac joint (*arrowhead*). Although the bone marrow edema in the right SI joint is subtle and not sufficient to fulfill ASAS criteria, it demonstrates that bone marrow edema can occur in the two locations in one patient and that these two lesions are not likely to be confused. **c** Unilateral LSTV grade II (*black arrow*)

As BME in SI joints is a hallmark of active axSpA and part of the ASAS classification criteria [1, 2], other entities causing BME may therefore mimic axSpA, as has been previously hypothesized [18]. Some LSTV patients showed BME at the enlarged transverse process and the portion of the sacral bone lying adjacent to it. BME was subtle and was confined to the immediate surfaces involved. Patients from all subtypes were represented in the BME group, but highest BME prevalence was found in grade II and IV patients (i.e. unilateral or bilateral pseudoarticulation), leading to the suggestion that pseudoarticulation, without proper alignment of surfaces, is more likely to cause reactive changes with concomitant BME (Fig. 2). The articulating site of the transverse process to the sacrum is in the upper quadrant of the sacrum, and BME at this level did not extend to the subchondral area of the SI joints in any of the patients. Typical axSpA BME lesions are located in the subchondral and periarticular area of the SI joint [15]. In addition, the signal intensity of the inflammation seen at the site of the (pseudo-) articulation was generally low and in contrast with the brighter BME lesions typically associated with axSpA. Therefore, we argue that BME caused by LSTV is unlikely to be misinterpreted as BME associated with axSpA. In addition, the presence of an LSTV is not associated with a higher prevalence of inflammation in the upper quadrants of the SI joints and the SI joints as a whole, as scored by axSpA readers. This means a (mechanical) correlation between an LSTV and inflammation in the SI joints is unlikely and thus an LSTV does not alter the MRI manifestation of axSpA.

As the LSTV alters the anatomy of the pelvic region, it also alters the distribution of (gravitational) forces [19]. In a normal spine, the L5–S1 vertebral unit (being the caudal-most, flexible part of the spine) is subject to the largest forces and therefore has the highest prevalence of degenerative changes reported [20]. As suggested before, with an LSTV, the intervertebral disc between the last lumbar and the first sacral vertebral body is “protected” and this may lead to increased degeneration of the penultimate disc [19, 21, 22]. We were able to confirm in our study that the prevalence of degeneration was comparable in no-LSTV and LSTV patients at L4–L5, was higher in no-LSTV patients in L5–S1 compared with L4–L5 and was lower in LSTV patients at L5–S1 than L4–L5 (Table 2), although differences were small and not statistically significant.

The association between LSTV and LBP, sometimes referred to as Bertolotti’s syndrome [8, 23], is controversial. LSTV types II and IV and unilateral presence have been reported to be associated with back pain [5, 7, 8], whereas other authors have not found an association between LBP and LSTV [3, 22]. We were unable to confirm the association between LSTV and LBP, as more than 90% of all patients in our cohort experienced LBP at inclusion, because back pain was the main inclusion criterion for the SPACE cohort. However, VAS pain scores were similar in the no-LSTV and LSTV groups. Spinal mobility (both lateral flexion and anterior flexion of the lumbar spine) was not different in the presence or absence of an LSTV, leading to the conclusion that these clinical parameters associated with axSpA were not different in the presence of an LSTV.

### Strengths

The SPACE cohort is a prospective observational study cohort, comprising young patients (age range 16–45) with short-term chronic back pain (more than 3 months, less than 2 years) and with suspected axSpA. Baseline radiographs and MRI scans were performed in all patients using standardized protocols, resulting in uniform imaging studies. At baseline (and subsequent visits) all patients were seen by a rheumatologist and investigator, leading to well documented clinical information. Because patients are included based on back pain rather than a diagnosis of axSpA, both patients with a diagnosis and those without a diagnosis are represented in the cohort.

### Limitations

The study population in our study does not represent the general population. All patients included in the SPACE cohort had had back pain for at least 3 months; thus, no symptom-free control population was available. Therefore, we were not able to make statements about radiological and clinical parameters associated with LSTV in the general population.

According to a study reported in 2014, coronal MRI is the superior method for the detection and classification of LSTV, with a higher reliability compared with standard radiographs [24]. However, standard AP radiographs were used in the current study; yet, inter-reader agreement was substantial and in the case of disagreement between the two readers, adjudication was performed by a third reader. Lastly, the small size of the LSTV subtype groups does not allow a comparative statistical analysis among individual subtypes, but numerical differences among LSTV subtypes were small (Tables 2, 3). Owing to the absence of weight-bearing full spine radiographs, an association of unilateral LSTV with scoliosis could not be assessed.

## Conclusion

The prevalence of LSTV was high in the SPACE cohort, which is in accordance with the literature. Although local BME was found only in patients with an LSTV and was absent in patients without LSTV, its presence in a fixed location is not likely to be confused with subchondral BME of the SI joints typically associated with axSpA. Furthermore, prevalence of LSTV did not differ between patients with and those without axSpA; radiological signs of axSpA were similarly present in no-LSTV and LSTV patients, and the results of clinical assessments were comparable among LSTV groups. Therefore, the presence of an LSTV has no clinical and radiological significance in diagnosing axSpA and in the symptoms of patients with suspected axSpA.

## References

[1] Rudwaleit M, van der Heijde D, Landewe R (2009). The development of Assessment of SpondyloArthritis international Society classification criteria for axial spondyloarthritis. II. Validation and final selection. Ann Rheum Dis.

[2] Rudwaleit M, van der Heijde D, Landewe R (2011). The Assessment of SpondyloArthritis International Society classification criteria for peripheral spondyloarthritis and for spondyloarthritis in general. Ann Rheum Dis.

[3] Tini PG, Wieser C, Zinn WM (1977). The transitional vertebra of the lumbosacral spine: its radiological classification, incidence, prevalence, and clinical significance. Rheumatol Rehabil.

[4] Castellvi AE, Goldstein LA, Chan DP (1984). Lumbosacral transitional vertebrae and their relationship with lumbar extradural defects. Spine.

[5] Tang M, Yang X, Yang S (2014). Lumbosacral transitional vertebra in a population-based study of 5860 individuals: prevalence and relationship to low back pain. Eur J Radiol.

[6] Apazidis A, Ricart PA, Diefenbach CM, Spivak JM (2011). The prevalence of transitional vertebrae in the lumbar spine. Spine J.

[7] Nardo L, Alizai H, Virayavanich W (2012). Lumbosacral transitional vertebrae: association with low back pain. Radiology.

[8] Quinlan JF, Duke D, Eustace S (2006). Bertolotti’s syndrome. A cause of back pain in young people. J Bone Joint Surg (Br).

[9] van den Berg R, de Hooge M, van Gaalen F, Reijnierse M, Huizinga T, van der Heijde D (2013). Percentage of patients with spondyloarthritis in patients referred because of chronic back pain and performance of classification criteria: experience from the Spondyloarthritis Caught Early (SPACE) cohort. Rheumatology.

[10] Van der Heijde D, Landewé R, Feldtkeller E (2008). Proposal of a linear definition of the Bath Ankylosing Spondylitis Metrology Index (BASMI) and comparison with the 2-step and 10-step definitions. Ann Rheum Dis.

[11] Sieper J, Rudwaleit M, Baraliakos X (2009). The Assessment of SpondyloArthritis international Society (ASAS) handbook: a guide to assess spondyloarthritis. Ann Rheum Dis.

[12] Pfirrmann CW, Metzdorf A, Zanetti M, Hodler J, Boos N (2001). Magnetic resonance classification of lumbar intervertebral disc degeneration. Spine.

[13] Fardon DF, Milette PC (2001). Nomenclature and classification of lumbar disc pathology. Recommendations of the combined task forces of the North American Spine Society, American Society of Spine Radiology, and American Society of Neuroradiology. Spine.

[14] Modic T, Steinberg M, Ross S, Carter R (1988). Degenerative disk disease: assessment of changes in vertebral body marrow with MR imaging. Radiology.

[15] Rudwaleit M, Jurik AG, Hermann KG (2009). Defining active sacroiliitis on magnetic resonance imaging (MRI) for classification of axial spondyloarthritis: a consensual approach by the ASAS/OMERACT MRI group. Ann Rheum Dis.

[16] Maksymowych WP, Inman RD, Salonen D (2005). Spondyloarthritis Research Consortium of Canada magnetic resonance imaging index for assessment of spinal inflammation in ankylosing spondylitis. Arthritis Rheum.

[17] Landis JR, Koch GG (1977). The measurement of observer agreement for categorical data. Biometrics.

[18] Padula A, Barozzi L, Ciancio G, Cantini F, Salvarani C, Olivieri I (1999). Involvement of transitional lumbosacral joints in spondyloarthritis. Clin Exp Rheumatol.

[19] Farshad-Amacker NA, Herzog RJ, Hughes AP, Aichmair A, Farshad M (2015). Associations between lumbosacral transitional anatomy types and degeneration at the transitional and adjacent segments. Spine J.

[20] Teraguchi M, Yoshimura N, Hashizume H (2014). Prevalence and distribution of intervertebral disc degeneration over the entire spine in a population-based cohort: the Wakayama Spine Study. Osteoarthritis Cartilage.

[21] Vergauwen S, Parizel PM, van Breusegem L (1997). Distribution and incidence of degenerative spine changes in patients with a lumbo-sacral transitional vertebra. Eur Spine J.

[22] Luoma K, Vehmas T, Raininko R, Luukkonen R, Riihimäki H (2004). Lumbosacral transitional vertebra: relation to disc degeneration and low back pain. Spine.

[23] Elster AD (1989). Bertolotti’s syndrome revisited. Transitional vertebrae of the lumbar spine. Spine.

[24] Farshad-Amacker NA, Lurie B, Herzog RJ, Farshad M (2014). Interreader and intermodality reliability of standard anteroposterior radiograph and magnetic resonance imaging in detection and classification of lumbosacral transitional vertebra. Spine J.

